# Myeloid sarcoma of the breast: a pathology that should not be forgotten

**DOI:** 10.3332/ecancer.2020.1160

**Published:** 2020-12-21

**Authors:** Luca Nicosia, Antuono Latronico, Mariagiorgia Farina, Anna Carla Bozzini, Paola Baratella, Viviana Enrica Galimberti, Stefano Fiori, Marta Montesano, Enrico Cassano

**Affiliations:** 1 Department of Breast Radiology, European Institute of Oncology, 20141 Milan, Italy; 2 Division of Breast Surgery, IEO, European Institute of Oncology, 20141 Milan, Italy; 3 Division of Pathology and Laboratory Medicine, IEO, European Institute of Oncology, 20141 Milan, Italy; 4 Department of Radiological Sciences, Oncology and Pathology, I.C.O.T. Hospital, ‘Sapienza’ University of Rome, 04100 Latina, Italy

**Keywords:** myeloid sarcoma, breast, biopsy, breast cancer

## Abstract

Myeloid sarcoma (MS) is a rare neoplasm, represented by a tumoural mass composed of myeloid blasts, occurring at any anatomical site other than the bone marrow. MS is considered the tissue-based equivalent of acute myeloid leukaemia (AML), requiring the same therapeutic specification, independently from the association with previous or coexisting myeloid neoplasms. Isolated breast involvement by MS is exceedingly rare, with only exceptional cases reported in the literature. This work aims to provide a pictorial essay of the main features of breast involvement by MS. Even though it is a rare condition, we should not forget this neoplasm, and its possibility of being disguised by the AML, as it requires prompt treatment.

## Introduction

Myeloid sarcoma (MS) was first described by Burns in 1811 and subsequently linked to myeloid neoplasms in 1893, by Dock [1]. MS is defined in the latest World Heath Organization (WHO) Classification of Tumours of Haematopoietic and Lymphoid Tissues as a tumour mass, with tissue effacement, composed of myeloid blasts, occurring at any anatomical site other than the bone marrow [2–4].

MS can variably precede or coincide with acute myeloid leukaemia (AML), represent relapse of a previous AML, or blastic transformation of myelodysplastic syndrome, myelodysplastic/myeloproliferative neoplasm or myeloproliferative neoplasm. A diagnosis of MS is hence considered equivalent to AML [5–7]. In the largest published series, MS occurring independently from AML or myeloid neoplasm is reported in about a quarter of the cases [6].

The most common sites involved are skin, lymph nodes, intestinal tract, bone and central nervous system; on the contrary, isolated breast involvement is exceedingly rare, with only exceptional cases reported in the literature. Histologically, MS is composed of myeloid blasts with the presence or the absence of neutrophilic or granulocytic maturation. Monocytic differentiation is quite common, while erythroid of megakaryoblastic features are uncommon and associated with transformation from myeloproliferative neoplasm. The major differential in diagnosis is mainly related to lymphoma and small round cell tumours, but even with poorly differentiated carcinoma is mandatory an immunohistochemical assessment, for a diagnosis of MS. Remarkably, 10 out of 25 isolated MSs have been misdiagnosed as diffuse large B-cell lymphoma (DLBCL) in the widest series [6].

Specific genetic alterations, such as t (8;21) (q22;q22.1) RUNX1-RUNXT1, inv (16) (p13.1q22) or t(16;16)(p13.1;q22) CBFB-MYH11, and NPM1 mutation, are linked to MS and extramedullary presentation of AML [7–9].

Recognising such alterations is crucial for the assessment of stratified risk and the subsequent therapeutic approach [8]. In isolated MS cases, without bone marrow involvement, such analysis may be problematic and require a fresh tissue sample to be submitted for cytogenetic or molecular procedures.

This work’s purpose is to provide a pictorial essay on the main features of breast MS. For the aggressiveness of this disease, it is important to initially consider such infrequent diagnosis, in order to start the correct diagnostic-therapeutic approach in a timely manner (including fresh tissue sampling to submit for cytogenetic and molecular analyses).

### Clinical appearance and presentation

The most common feature of MS is the presence of a rapidly growing mass: clinically, MS involving the breast can present as a unilateral or bilateral mass and often can be indistinguishable from benign tumours or lymphoma.

### Radiological and histopathological appearance

**Figure 1.**
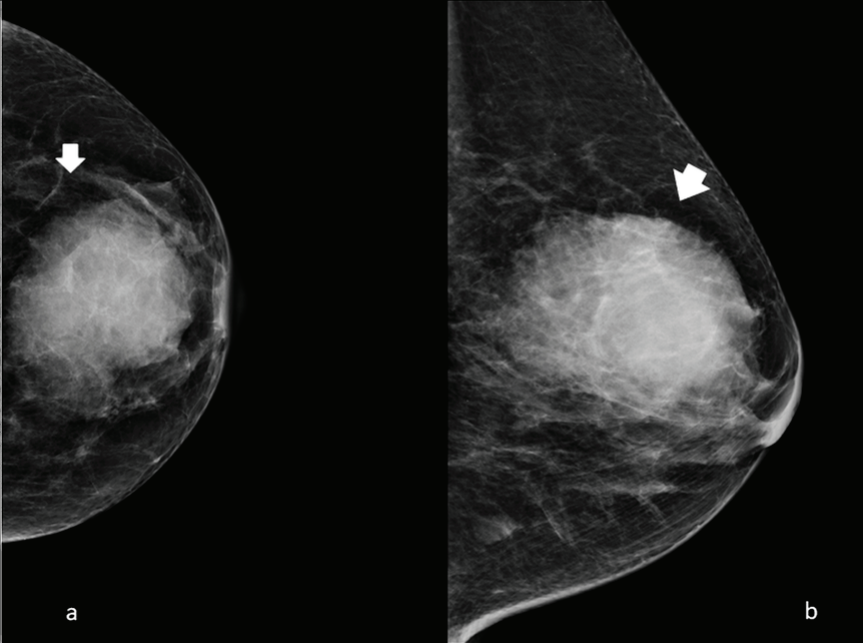
Left side full-field digital mammography of a 70-year-old patient presenting a unilateral breast mass subsequently identified as a MS. (a): Cranio caudal and (b): mediolateral oblique projection, showing a noncalcified ill-defined radiopaque mass with poorly defined ‘feathery’ margins (arrow).

**Figure 2.**
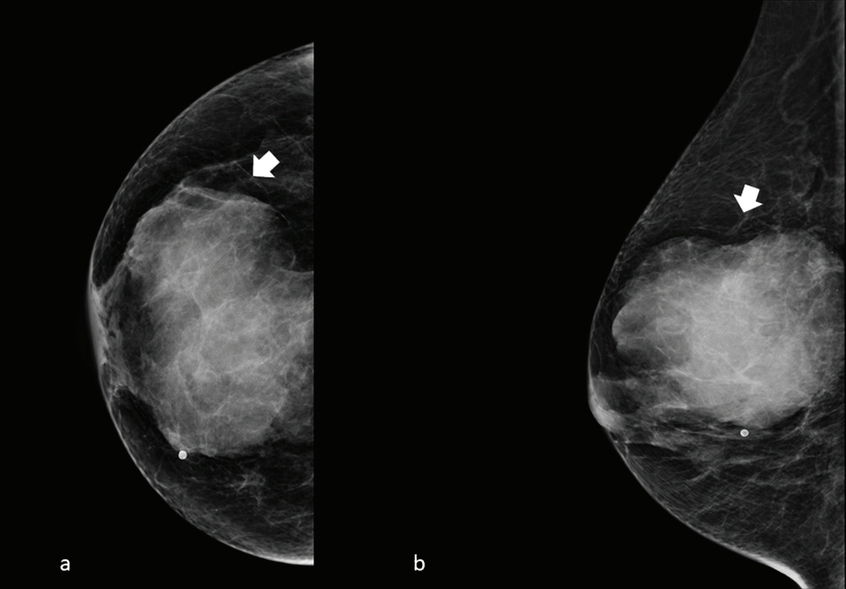
Right side full-field mammography of a 40-year-old patient presenting a unilateral breast mass subsequently identified as a MS. (a): Cranio caudal and (b): mediolateral oblique projection, showing a noncalcified ill-defined radiopaque mass with poorly defined ‘feathery’ margins (arrow).

The typical mammogram manifestation of granulocytic sarcoma is the presence of a large, non-calcified irregular mass with poorly defined ‘feathery’’ margins [10, 11], as shown in Figures 1 and 2.

**Figure 3.**
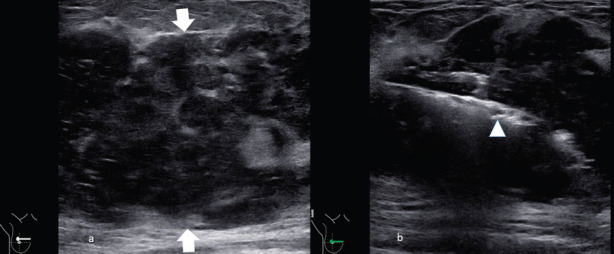
A 55-year-old patient presenting a right unilateral breast mass subsequently identified as a MS. (a): The heterogeneous echo patterns and marked low attenuation, with ill-defined margins (arrow). (b): The biopsy of the lesion with the needle inside the lesion (arrowhead).

**Figure 4.**
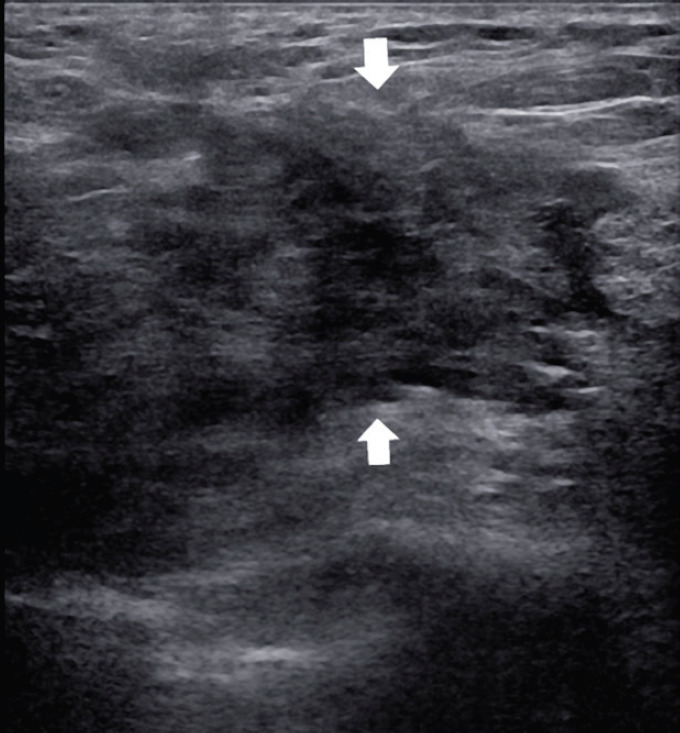
A 60-year-old patient presenting a right unilateral breast mass subsequently identified as a MS. The heterogeneous echo patterns and marked low attenuation are appreciated, with ill-defined margins (arrows).

Frequently the diagnosis is performed with ultrasound biopsy [12, 13]: heterogenous echo patterns and marked low attenuation, with ill-defined margins, are the typical ultrasound features as shown in Figures 3 and 4.

**Figure 5.**
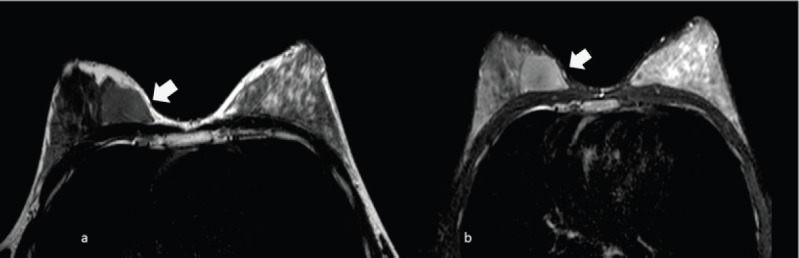
A 70-year-old patient presenting a right unilateral breast mass subsequently identified as a MS. (a): Ill-defined, heterogeneous, hyperintense masses relative to breast parenchyma on T2 images (arrow) and (b): hypointense on T1 images (arrow).

**Figure 6.**
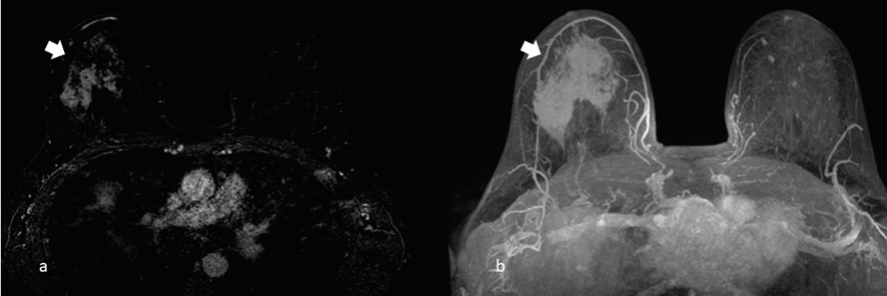
A 63-year-old patient presenting a right unilateral breast mass subsequently identified as a MS. (a) Inhomogeneous enhancement on gadolinium administration (arrow) and (b): the appearance of the mass enhancement (arrow) in the maximum intensity projection.

**Figure 7.**
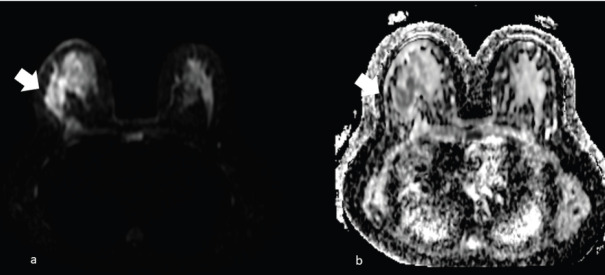
The same patient of Figure 6, showing right unilateral breast mass, subsequently identified as a MS. (a): Diffusion imaging with hyperintense mass (arrow) and (b): the restricted diffusion of the same mass (arrow) in the apparent diffusion coefficient (ADC) mass.

Magnetic resonance imaging using T2-weighted images may show granulocytic sarcoma as ill-defined, heterogeneous, hyperintense masses relative to breast parenchyma, and hypointense on T1 images (Figure 5), inhomogeneous enhancement (Figure 6) on gadolinium administration and restricted diffusion (Figure 7).

**Figure 8.**
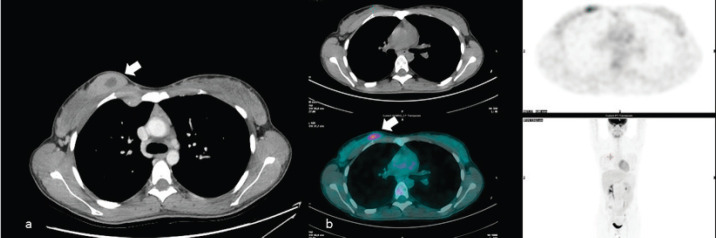
A 50-year-old patient presenting a right unilateral, incidental finding of breast mass subsequently demonstrated to be a MS. (a): Irregular breast mass on the computed tomography exam, showing inhomogeneous contrast enhancement (arrow). (b):The abnormal glucose uptake in PET examination (arrow).

MS can also be found fortuitously during CT or PET examination in oncologic patients ([14–16]; Figure 8).

**Figure 9.**
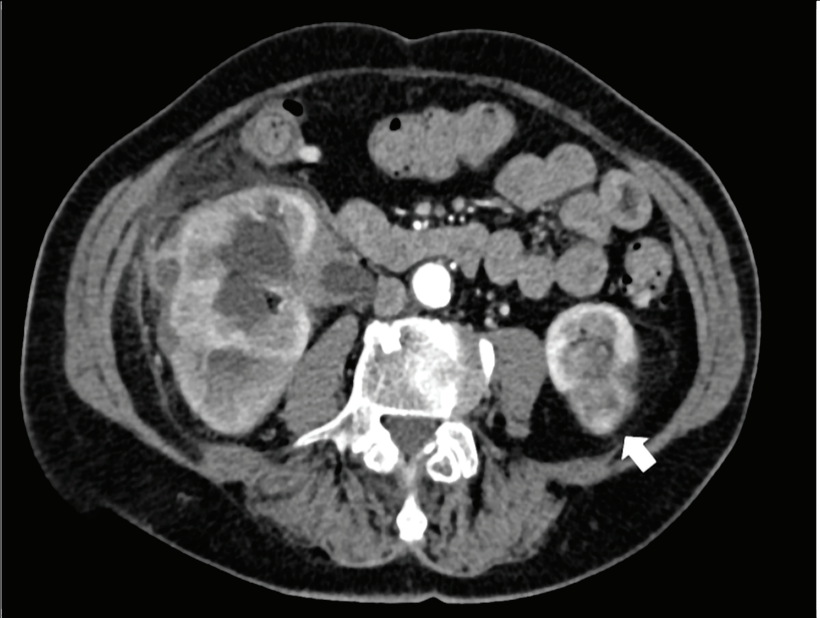
Same patient of Figure 8, showing irregular contrast-enhancing soft tissue mass (arrow), resulting in the localisation of MS.

**Figure 10.**
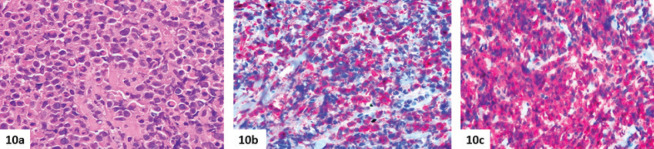
Histologic features of breast MS. (a–c): Breast involvement by MS with inv(16) (p13.1q22) in a 21-year-old female patient. Myeloid blasts are associated with eosinophils (a, haematoxylin-eosin) and express CD34 (b) and myeloperoxidase (c). In this case, the disease involvement was limited to the right breast, and a second needle biopsy was required to assess cytogenetic and molecular features.

**Figure 11.**
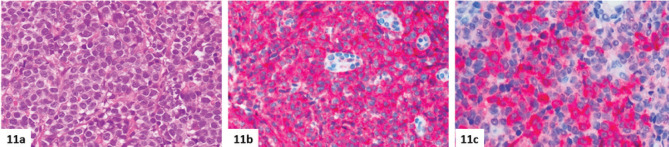
(a–c): Breast involvement by MS in a 76-year-old female patient. The neoplasm is made up of solid sheets of large blastic cells without maturation (a, haematoxylin-eosin), positive for CD34 (b) and myeloperoxidase (c). In this case, the disease arose with bilateral breast masses, but disseminated involvement (mammary, central nervous system, renal, cardiac, uterine, skeletal and nodal PET-positive lesions, massive bone marrow infiltration) was then discovered. Cytogenetic and molecular analyses, performed on marrow aspirate, did not show any alterations, and a diagnosis of acute myeloid leukaemia without maturation, with extramedullary involvement/MS was made.

**Figure 12.**
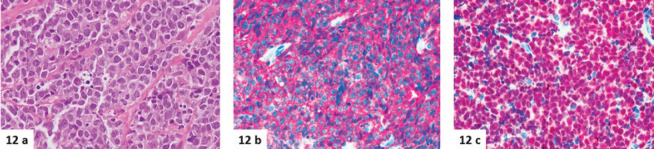
(a–c): A case of DLBCL, as an isolated left breast lesion in a 57-year-old woman. The lesion is made up of solid sheets of large lymphoid blastic cells (a, haematoxylin-eosin), quite similar to the myeloid blasts shown in Figures 10a and 11a. Diffuse positivity for CD20 (b) and PAX5 (c) proves the B-cell lineage of the neoplasm, but extensive immunohistochemical analyses are mandatory to differentiate MS from aggressive lymphomas.

The typical appearance is of an irregular breast mass with inhomogeneous contrast enhancement and glucose uptake. On occasion, patients with MS of the breast can present other soft tissue localisation, such as the kidney (Figure 9). The histopathological diagnosis is challenging especially for the difficulties encountered distinguishing MS from lymphoma (Figures 10–12).

## Conclusion

The MS of the breast tissue, with no signs of leukaemia, is extremely rare [17, 18]: providing a correct diagnosis can be challenging for the breast pathology team, as it is crucial to decide the best therapeutic approach. MS is an aggressive disease, for it is the solid counterpart of AML, and is treated accordingly, eventually requiring allogeneic bone marrow transplantation to achieve complete remission. For this reason, and for the objective diagnostic complexity in isolated MS cases, the identification of characteristic imaging features could be very useful. This pictorial review’s purpose is to provide a showcase of some of the most important imaging and pathologic features that allow the confrontation and the diagnosis of this particular and extremely rare type of neoplasm. Besides being rare, this tumour closely mimics other neoplasms; it is, therefore, important to be aware of this pathology and its main radiological and anatomopathological characteristics.

## Conflict of interest

All authors declare that they have no conflict of interest.

## Funding

This research did not receive any specific grant from funding agencies in the public, commercial or not-for-profit sectors.
